# Phylogeography and demographic history of *Lacerta lepida *in the Iberian Peninsula: multiple refugia, range expansions and secondary contact zones

**DOI:** 10.1186/1471-2148-11-170

**Published:** 2011-06-17

**Authors:** Andreia Miraldo, Godfrey M Hewitt, Octavio S Paulo, Brent C Emerson

**Affiliations:** 1 School of Biological Sciences, University of East Anglia, Norwich, NR4 7J, UK; 2 Centro de Biologia Ambiental/Departamento de Biologia Animal, Faculdade de Ciencias da Universidade de Lisboa, P-1749-016 Lisboa, Portugal; 3 Metapopulation Research Group, University of Helsinki, Department of Biological and Environmental Sciences, P.O. Box 65, 00014 Helsinki, Finland; 4 Island Ecology and Evolution Research Group, IPNA-CSIC, C/Astrofísico Francisco Sánchez 3, 38206 La Laguna, Tenerife, Canary Islands, Spain

## Abstract

**Background:**

The Iberian Peninsula is recognized as an important refugial area for species survival and diversification during the climatic cycles of the Quaternary. Recent phylogeographic studies have revealed Iberia as a complex of multiple refugia. However, most of these studies have focused either on species with narrow distributions within the region or species groups that, although widely distributed, generally have a genetic structure that relates to pre-Quaternary cladogenetic events. In this study we undertake a detailed phylogeographic analysis of the lizard species, *Lacerta lepida*, whose distribution encompasses the entire Iberian Peninsula. We attempt to identify refugial areas, recolonization routes, zones of secondary contact and date demographic events within this species.

**Results:**

Results support the existence of 6 evolutionary lineages (phylogroups) with a strong association between genetic variation and geography, suggesting a history of allopatric divergence in different refugia. Diversification within phylogroups is concordant with the onset of the Pleistocene climatic oscillations. The southern regions of several phylogroups show a high incidence of ancestral alleles in contrast with high incidence of recently derived alleles in northern regions. All phylogroups show signs of recent demographic and spatial expansions. We have further identified several zones of secondary contact, with divergent mitochondrial haplotypes occurring in narrow zones of sympatry.

**Conclusions:**

The concordant patterns of spatial and demographic expansions detected within phylogroups, together with the high incidence of ancestral haplotypes in southern regions of several phylogroups, suggests a pattern of contraction of populations into southern refugia during adverse climatic conditions from which subsequent northern expansions occurred. This study supports the emergent pattern of multiple refugia within Iberia but adds to it by identifying a pattern of refugia coincident with the southern distribution limits of individual evolutionary lineages. These areas are important in terms of long-term species persistence and therefore important areas for conservation.

## Background

Evidence from phylogeographic studies suggests that southern Europe, including the peninsulas of Iberia, Italy and the Balkans and areas near the Caucasus and the Caspian Sea, have functioned as refugial areas for species survival during periods of adverse climatic conditions [1]. Recent studies also emphasize the important role that these refugial areas had in shaping the evolutionary history of species that have persisted within these regions for several ice ages. As suggested by early studies [2-4] the topographic complexity and geographic mosaic of habitats in southern refugial peninsulas have favoured the occurrence of multiple disjunct refugia, allowing the persistence of isolated populations within them during glacial periods. Within the Iberian Peninsula, complex species histories have been revealed for a variety of taxa, with some showing remarkable patterns of phylogeographic concordance [see [5] and references therein] involving deep genetic subdivisions, high haplotype richness and distinct hybrid zones. Not only has the Iberian Peninsula sourced the northern redistribution of species after ice ages, but it has also facilitated diversification through patterns of repeated population fragmentation, contraction, expansion and admixture. Phylogeographic analyses for the golden-striped salamander, *Chioglossa lusitanica *[6-8] and Schreiber's Lizard, *Lacerta schreiberi *[9,10] provide good examples of the complexity that most likely typifies many species within this major peninsular glacial refugium.

However, even though the Iberian Peninsula is the best studied glacial refugium, most phylogeographic studies have focused on species that either have a narrow distribution within the region (e.g *Chioglossa lusitanica *[6], *Lacerta schreiberi *[9], *Lissotriton boscai *[11]) or involve species groups that, although distributed across the entire region, generally have a genetic structure that relates to older cladogenetic events (e.g *Podarcis *spp. [12,13], *Alytes *spp [14], *Oryctolagus cunniculus *[15,16]). In order to better understand the complex phylogeographic history of Iberian species, and the way they have responded to Pleistocene climatic oscillations, it is important to study species with distributions encompassing the entire Iberian Peninsula. For this purpose we have used the ocellated lizard, *Lacerta lepida *(Daudin, 1802), as a model to study the impact of Pleistocene climatic changes in generating and structuring intraspecific genetic diversity on this regional scale. *Lacerta lepida *is typically Mediterranean, with a distribution encompassing all the Iberian Peninsula, and with phylogeographic structure across the region [17]. Several mitochondrial lineages that appear to have non-overlapping geographic ranges were recently described, suggesting a history of allopatric differentiation in multiple refugia during the Plio-Pleistocene [17]. Here we assess the broader phylogeographic patterns within *Lacerta lepida *with the specific aims to i) clarify the distribution of mtDNA phylogroups; ii) identify refugial areas within these phylogroups during the glacial periods; iii) date the main demographic and evolutionary events within *Lacerta lepida*; and finally iv) identify secondary contact zones between the different phylogroups.

It is generally accepted that phylogeographic histories recovered using only mtDNA as a marker are constrained to reveal one genealogy that reflects the maternally inherited natural history of an organism. Relationships among phylogroups inferred through mtDNA might be discordant with inferences made based on nuclear genes (Harrison 1991; Avise 2000) and discordances have been illustrated in several studies [e.g. [18-24]]. Particularly within the Iberian Peninsula, several studies have emphasized the importance of using different types of markers to fully recover the complex evolutionary and demographic scenarios that most likely characterize the species that have persisted there across the Quaternary. For example, in *Lacerta schreiberi *evidence for gene flow and ancestral introgression between apparently allopatric mtDNA lineages was only detected by the use of nuclear markers [10]. Here we use both mtDNA and nDNA derived genealogies, since their contrasting molecular and population properties (principally uniparental versus biparental mode of inheritance and contrasting population sizes) are mutually informative when there have been opportunities for secondary contact, gene flow and hybridization between diverging populations.

## Methods

### Sampling

Lizards were captured under licence during the years 2005, 2006 and 2007, sampling most of the distribution of *Lacerta lepida *in Portugal, Spain, and France. Sampling intensity was concentrated in regions known to contain high genetic divergence within western and south-eastern part of Iberia [17]. Lizards were captured using tomahawk traps or by hand, and tissue samples were taken by clipping 1 cm of the tail tip that was subsequently preserved in 100% ethanol. After tissue sampling, animals were immediately released back into the wild in the place of capture. Geographic coordinates of sampling sites were recorded with a GPS.

### DNA extraction, amplification and sequencing

Total genomic DNA was extracted from ethanol-preserved muscle tissue using a salt extraction protocol [25,26]. A fragment of 627 base pairs (bp) of the mitochondrial DNA (mtDNA) cytochrome b (*cytb*) gene was amplified using the truncated version of primer L14841 [27] (CYTBF, 5'-CCA TCC AAC ATC TCA GCA TGA TGA AA-3') and the modified version of primer MV16 [28] (CYTBR, 5'- AAA TAG GAA GTA TCA CTC TGG TTT-3') to increase specificity for *Lacerta lepida*, using information from published sequences in GenBank. Polymerase chain reactions (PCRs) were performed in a total volume of 25 μl, containing 0.5 U of Taq polymerase (BIOTAQ™), 4 mM of MgCl_2_, 0.4 mM of each nucleotide (Bioline), 0.4 μM of each primer, 2 μl of 10 × NH_4 _reaction buffer (Bioline) and approximately 50 ng of DNA. PCR amplifications were conducted as follows: DNA was initially denaturated at 94°C for 3 min followed by 35 cycles of denaturation at 94°C for 45 s, annealing at 51°C for 45 s and extension at 72°C for 45 s, plus a final extension step at 72°C for 5 min. Negative controls (no DNA) were included for all amplifications. PCR products were visualized on a 2% agarose gel and purified by filtration through QIAquick^® ^columns (Qiagen) following the manufacturer's recommendations. Purified PCR products were sequenced in both directions using the above primers, Taq polymerase BigDye Terminator v3.1™ (Applied Biosystems) and 30-90 ng of PCR product.

Intron 7 of the β-fibrinogen gene (*β-Fibint7*) has been successfully used as a nuclear marker in several vertebrate phylogeographic and phylogenetic studies [e.g. [13,29-32]]. Specifically it was recently employed for a phylogenetic study of the genus *Lacerta *[17] where it revealed sufficient variation within *Lacerta lepida *to be useful for phylogeographical inference. Initial *β-fibint7 *amplifications were performed using primers FIB-B17U (5'- GGA GAA AAC AGG ACA ATG ACA ATT CAC - 3') and FIB-B17L (5' - TCC CCA GTA GTA TCT GCC ATT AGG GTT - 3') [29] and the conditions described in Paulo *et al*. [17]. However, due to low amplification and sequencing success a nested PCR approach as suggested by Sequeira *et al*. [32] was subsequently adopted. A fragment of 788bp was first amplified from genomic DNA using primers FIB-B17U and FIB-B17L (PCRa). The product of this reaction (1 μl) was then used as a template for a subsequent PCR of 691bp (PCRb) using primer BFXF [32] and BFX8 [13]. Both amplifications were performed in a total volume of 25 μl, and included reagents in the same concentrations as those specified for *cytb *gene fragment. PCR cycle conditions were the same as described for *cytb *fragment but the primer annealing temperatures were 55°C and 56°C, for PCRa and PCRb respectively. Negative controls (no DNA) were included for all amplifications. Purified PCRb products were then sequenced with primers BFBX and BFX8 using identical sequencing conditions as for the mtDNA *cytb *sequencing. All PCRs and sequencing reactions were performed in a MJ Research thermocycler (PTC-240 DNA Engine Tetrad 2) and sequences were obtained using an ABI 3700 capillary sequencer.

### Sequence analysis

DNA sequences were aligned by eye using BioEdit Sequence Alignment Editor 7.01 [33]. *β-fibint7 *alleles of heterozygous individuals were inferred using Phase version 2.1 [34,35]. Ten replicate Phase runs were conducted using 1000 burnin steps and 1000 iterations. The Phase probability threshold was first set to 0.90, but not all genotypes were resolved at this threshold. Garrick *et al*. [36], suggest that lowering the Phase threshold to 0.60 reduces the number of unresolved haplotypes with little or no increase in the number of false positives. In contrast, omitting unresolved haplotypes may have a substantial impact on downstream analyses, such as the estimation of Tajima's *D *and Fu's *Fs *statistics, as unresolved haplotypes usually represent rare alleles [36]. We therefore reduced the Phase threshold to 0.60 to maximise recovery of haplotypes. Several tests implemented in the software RDP3 [Recombination Detection Program, [37]] were used to detect evidence for recombination in *β-fibint7*: RDP [38], GENECONV [39], Maximum Chi Square [40,41], Chimaera [40] and Sister Scanning [42]. Due to the small size of the fragment used in the analyses (315 bp), the window size for the recombination detection methods was set to 20bp.

### Phylogenetic analysis and haplotype network construction

Phylogenetic relationships among *cytb *haplotypes were inferred using Bayesian inference as implemented in Mrbayes v3.1.2 [43,44]. Data was divided into two partitions: (1^st^+2^nd^) and (3^rd^) codon positions. The most appropriate substitution model for each partition was selected using Modeltest v3.7 [45] and the Akaike Information Criterion (AIC) [46]. The model selected for both partitions was the same (GTR + I) but with a different proportion of invariable sites (pinv(1^st^+2^nd^) = 0.79 and pinv(3^rd^) = 0.09). The different partitions were allowed to evolve at different rates, with unlinked topology and unlinked parameters for the nucleotide substitution models. Four Markov chains were run for 10 million generations. To avoid local optima we used two independent runs, and to improve swapping of states between heated and cold chains the heating parameter was decreased to 0.05. Trees were sampled every 1000 generations and the first 100 trees were discarded as burn-in. Posterior probabilities were obtained from the 50% majority-rule consensus tree of the retained trees.

Intraspecific gene genealogies were inferred using the median-joining (MJ) [47] and the statistical parsimony (SP) [48] network construction approaches. The MJ network was computed with the program Network 4.5.0 (http://www.fluxus-engineering.com) and the SP network was inferred using the program Tcs 1.21 [49]. For the MJ approach the parameter *ε *was set to 0, preventing less parsimonious pathways from being included in the analysis. The SP network was inferred with a parsimony confidence limit of 95%, allowing the inclusion of less parsimonious alternatives that fall within this confidence limit. Ambiguities within networks were resolved where possible following the criteria of Crandall & Templeton [50]. The phylogenetic and *β-Fibint7 *network analyses were rooted using gene sequences of the African sister species *Lacerta pater *(Lataste, 1880) [17,51] (Genebank accession numbers: AF: 378967 and EU: 365413 for *cytb *and *β-Fibint7 *genes respectively).

### Estimation of divergence times

Divergence times within and between phylogroups were estimated using Beast version 1.4.8 [52] and the *cytb *dataset. Beast performs Bayesian statistical inferences of parameters, such as divergence times, by using Markov Chain Monte Carlo (MCMC) as a framework. Input files were generated with Beauti version 1.4.8 [53]. The nucleotide substitution model and its parameter values were selected according to the results of Modeltest version 3.7 [45], with upper and lower bounds around the values defined as 120% and 80% respectively [54]. According to the Bayesian Information Criterion (BIC) and the hierarchical Likelihood Ratio Tests (hLRT's), the model of nucleotide substitution identified as the best fit to the data is the HKY model [55] with a gamma distribution (Γ) for substitution rates across sites (shape parameter, α = 0.2889) and no category of invariable sites. An uncorrelated lognormal relaxed molecular clock was used with mean mutation rate of 0.01 mutations/site/million years and standard deviation of 0.0015, assuming a normal distribution, as prior information. The mutation rate used was based on the provisional calibration of a reptile-specific molecular clock presented in Paulo *et al*. [9] who used published cytochrome b data sets from two studies of Canary island reptiles (*Gallotia *spp.) [56,57] calibrated with the geological age of El Hierro. The standard deviation included in our study encompasses a faster mutation rate of 0.0115 and a slower mutation rate of 0.0085 mutations/site/million years. This takes into account potential underestimation of the mutation rate due to violation of the assumption of immediate island colonization in the clock calibration of Paulo *et al*. [9], or overestimation due to the longer generation time and larger body size of *Lacerta *spp. when compared to *Gallotia *spp. [58]. Two runs were executed for 10^6 ^generations, sampling every 500 generations and discarding the first 10% as burn in. Results of the two runs were displayed and combined in Tracer v1.4 [59] to check for stationarity and ensure that ESSs were above 200. For all analyses one sequence of *Lacerta pater *(Genebank accession number: AF378967) was included as an outgroup.

In a second approach to estimate divergence times within phylogroups the method of Saillard *et al*. [60] was employed where each extant haplotype descending from the most recent common ancestor (MRCA) represents a time interval between the present and the MRCA. Average distances from the MRCA within each phylogroup are calculated from the number of mutational steps separating each haplotype from the MRCA. The absolute timing of divergence is then calculated by multiplying the observed values by the average mutational changes per lineage per million years (Myr). Three mutation rates were used: 0.01 mutations/site/million years, representing the average mutation rate; a faster mutation rate of 0.0115, and a slower mutation rate of 0.0085.

### Neutrality tests and demographic analyses

In order to detect departures from a constant population size under the neutral model, Tajima's *D *[61], Ramos-Onsins & Rozas' *R*_*2 *_[62] and Fu's *Fs *[63] tests were applied to both mtDNA and nDNA datasets. Both *R*_*2 *_and *Fs *statistics have been shown to be the best statistical tests to detect population growth (*R*_*2 *_has been suggested to behave better for small sample sizes whereas *Fs *is better for bigger ones) [62]. Population expansions have also been shown to leave particular signatures in the distribution of pairwise sequence differences [64,65]. We capitalized upon this by employing statistics based on the mismatch distribution to test for demographic expansions. The observed distribution of pairwise differences between haplotypes within phylogroups was compared with the expected results under a sudden-demographic and a spatial-demographic expansion model. Statistically significant differences between observed and simulated expected distributions were evaluated with the sum of the square deviations (*SSD*) and Harpending's raggedness index (*hg*) [66,67]. For the *β-Fibint7 *gene we used two datasets: the first including only haplotypes inferred by Phase with probability higher than 0.90, and the second including all haplotypes inferred with the less stringent Phase probability threshold of 0.60. Tests were performed with Arlequin version 3.11 [68] for Tajima's *D*, Fu's *Fs*, *SDD *and *hg*, and with DNAsp version 4.50 [69] for *R*_*2 *_and expected values for the mismatch distribution. The demographic history of each phylogroup was also inferred using a coalescent-based approach. The model used to infer past population dynamics was the Bayesian Skyline Plot (BSP, [70]) as implemented in Beast version 1.4.8 [52]. For each phylogroup we carried out two independent runs of 10 million generations each. Trees and parameters were sampled every 1000 iterations, with a burn in of 10%. Results of each run were visualized using Tracer v1.4 [59] to ensure stationarity and convergence had been reached, and that effective sample sizes (ESS) were higher than 200.

### Geographical distribution of alleles and inference of refugial areas

Using predictions from coalescent theory (haplotypes at the tips of a tree are younger than interior haplotypes to which they are connected) ancestral and derived haplotypes within each phylogroup were identified, thus obtaining a temporal framework for inferring haplotype origin within phylogroups. The null hypothesis of random geographic distribution of haplotypes was tested statistically using Geodis version 2.5 [71] with significance testing by permuting the data 10^6 ^times. When non-random associations of haplotypes with geography were detected, the geographic distribution of ancestral versus derived haplotypes (interior and tip haplotypes, respectively) was further explored to identify possible refugial areas and the directionality of previously detected demographic and spatial expansions. Coalescent theory predicts refugial areas to exhibit a high frequency of ancestral haplotypes [72], and therefore the average number of mutations of a haplotype from the MRCA is expected to be significantly lower in refugial areas than would be expected by chance. In contrast, recently colonized areas are predicted to show a high incidence of derived haplotypes [72], and therefore the average number of mutations of a haplotype from the MRCA is expected to be significantly higher than would be expected by chance. To test the hypothesis that refugial areas are located in the south of a phylogroup's distribution, with the north having been more recently colonized, we estimated the average distances of alleles from the MRCA within both the north and south of the geographic range of a phylogroup. We then tested whether these values were significantly high or low by randomizing the geographic states of alleles to generate a null distribution of mean haplotype distance from the MRCA within both the south and north of a phylogroup's distribution.

## Results

**Figure 1:**
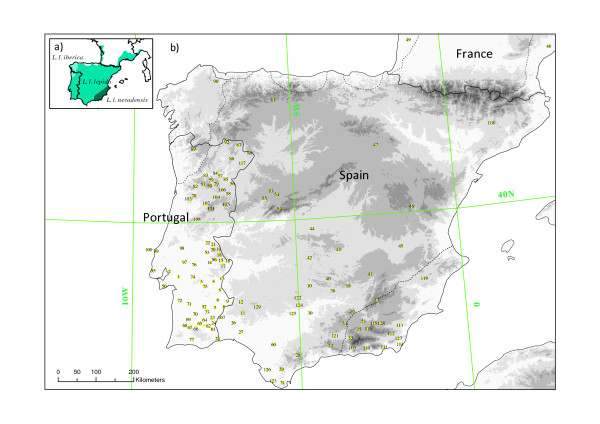
**Map of the Iberian Peninsula showing the current distribution of Lacerta lepida and sampled localities.** a) Distribution of the recognized continental subspecies of *Lacerta lepida *(*L. lepida. iberica, L. lepida lepida *and *L. lepida nevadensis*). b) Sampling localities. Numbers are as in Additional file 1, Table S1. Shaded areas denote altitude gradients, with darker areas representing higher altitudes.

A total of 353 lizards were sampled from 129 different sites across the distribution of *Lacerta lepida*. Sampling sites and number of samples per site are shown in Figure 1 and Additional file 1, Table S1, respectively. A total of 321 *cytb *and 104 *β-Fibint7 *sequences were obtained.

### Sequence variability

All *cytb *sequences represented uninterrupted open reading frames, with no gaps or premature stop codons, suggesting they are functional mitochondrial DNA copies. The chromatograms of 9 sequences were polymorphic at several nucleotide positions. These samples were from individuals collected at sampling sites 59 (1 sample), 79 (2 samples), 80 (1 sample), 84 (1 sample), 86 (2 samples), 88 (1 sample) and 89 (1 sample). As it was not possible to correctly identify the corresponding mitochondrial sequence of each sample these sequences were eliminated from further analysis. One hundred and forty five (145) unique haplotypes were obtained from the 312 sequences analysed. Of a total of 627 sites sequenced, 168 were variable, from which 129 were parsimony informative. Pairwise genetic distances (uncorrected p-values) between haplotypes ranged from 0.16% to 13.2% (Table 1). *β-fibint7 *sequences were trimmed to 315 bp in order to eliminate gaps within the sequences. From the 315 bp, 15 sites were variable of which 6 were parsimony informative. The haplotypes inferred by Phase for heterozygous individuals were consistent across runs. Twenty unique haplotypes (B1 to B20) were identified among the 208 alleles analysed and recombination was not detected by any of the tests applied. When using a Phase probability threshold of 0.90, 8 genotypes were unresolved (Additional file 1, Table S1), and this included the loss of only a single haplotype (B7) that was additionally recovered with a probability threshold of 0.60.

**Table 1:** Average pairwise genetic distances between *Lacerta lepida *mitochondrial phylogroups.

mtDNA phylogroup	L1	L2	L3	L4	L5	N
	uncorrected p distances (%)					
	
L1	**0.32 (0.55)**	3.28	2.53	1.48	1.85	11.30
L2	3.74	**0.55 (0.53)**	2.75	2.92	3.28	12.44
L3	2.80	3.13	**0.67 (0.64)**	2.10	2.44	12.02
L4	1.57	3.29	2.30	**0.74 (0.71)**	1.11	11.74
L5	2.00	3.77	2.72	1.17	**0.61 (0.59)**	11.86
N	19.07	22.38	21.20	20.61	21.07	**0.72 (0.69)**
	
	HKY + **Γ **corrected (%)					

Values in diagonal represent average pairwise genetic distances within phylogroups. Values above diagonal and in diagonal inside brackets represent uncorrected genetic distances (p distances) and values under diagonal and in diagonal outside brackets represent genetic distances corrected using the HKY + Γ model for nucleotide substitution.

### Phylogenetic and network relationships among haplotypes

**Figure 2:**
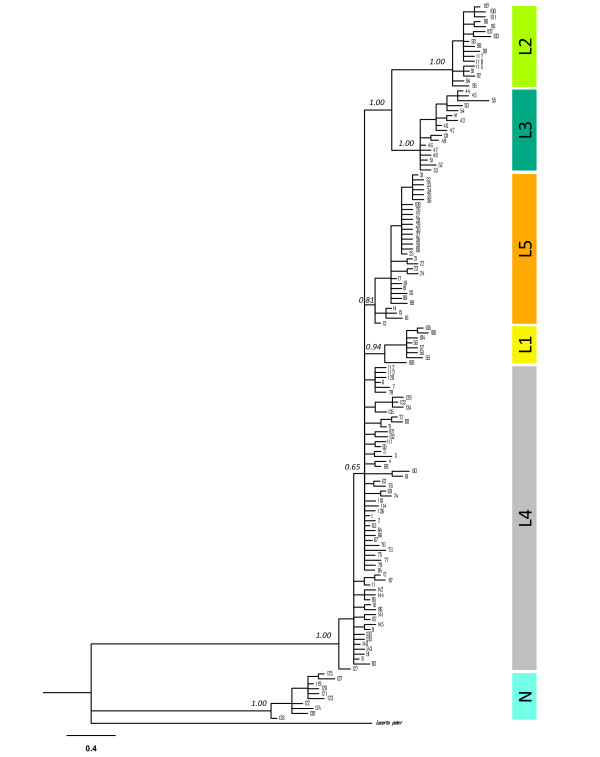
**Phylogenetic relationships among cytochrome b sequences (627 bp)**. Fifty percent majority-rule consensus phylogram from the Bayesian inference analysis. Numbers above branches represent posterior probabilities. Haplotype numbers are the same as in Figure 3 and as in Additional file, Table S1. Colours represent the geographic distribution of haplotypes as in Figure 5.

**Figure 3:**
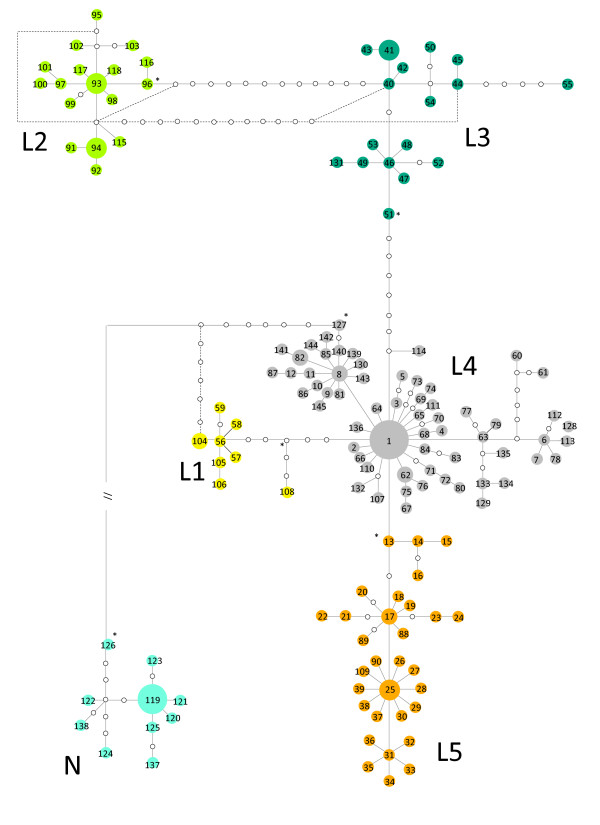
**Statistical Parsimony network of *Lacerta lepida *cytochrome b haplotypes from 312 samples**. Open circles with no numbers represent unsampled or extinct haplotypes. L1, L2, L3, L4, L5 and N represent different mitochondrial phylogroups. The ancestral haplotype within each phylogroup is marked with an asterisk. Phylogroup N connects to the main network through 65 mutations, represented by an interrupted line.

The results of the phylogenetic and network analyses both support the existence of two main phylogroups: phylogroup N and phylogroup L (Figure 2). The geographic distribution of phylogroup N is coincident with the Betic Mountains in south-eastern Spain and is associated with the subspecies *Lacerta lepida nevadensis *(Buscholz, 1963), while phylogroup L occupies the remaining area of the species distribution. Within phylogroup L there are four geographically structured monophyletic groups (L1, L2, L3 and L5), albeit with only moderate Bayesian support for the monophyly of L5 (Figure 2). The genealogical relationships among *cytb *haplotypes inferred by the two approaches for network construction (MJ and SP) are highly congruent. At the 95% confidence limit TCS produced three unconnected networks (results not shown). When the confidence limit was reduced (to 92% to include group L2 and to 65 mutational steps for group N) the three SP networks were connected in one single network (Figure 3), with relationships identical to those from the MJ analysis, with 12 loops, of which 7 were easily resolved by applying the criteria of Crandall and Templeton [50].

**Figure 4:**
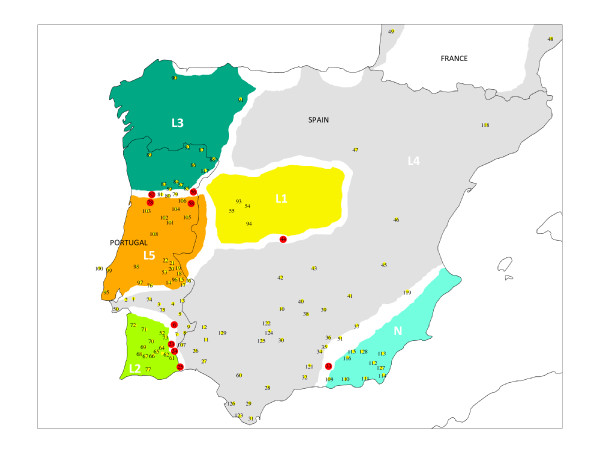
**Distribution of *Lacerta lepida *mitochondrial phylogroups based on 627 bp of the cytochrome b gene**. Colours are the same as in Figures 2 and 3. Filled red circles represent populations where divergent haplotypes from two or more phylogroups were detected in sympatry. Numbers correspond to sampling localities as in Figure 1 and Additional file 1, Table S1.

Within phylogroup L, five geographically distinct groups of haplotypes can be identified (Figure 3 and Figure 4), which include the four mitochondrial phylogroups (L1-L4) identified by Paulo *et al*. [17] and a new lineage of haplotypes (L5) possessing a geographically distinct distribution from other haplotypes within phylogroup L4. Average genetic distances (uncorrected p distances) between these phylogroups range from 1.1% (between phylogroup L4 and L5) to 3.28% (between phylogroups L2 and L5; and L2 and L1). Phylogroup L1 is distributed mainly across the Central Mountain system between the Douro and Tagus river basins in Spain. Phylogroup L2 is distributed in southern Portugal, occupying the regions of Algarve and Baixo Alentejo. This phylogroup is clustered together in the network with phylogroup L3 (Figure 3) forming a monophyletic group in the phylogeny (Figure 2). L3 is distributed several hundred kilometres (300 to 450 km) to the north of L2 occupying the north-western corner of Iberia, mainly the regions to the north of the Douro River in Portugal and the regions of Asturias and Galicia in Spain. The geographic region between phylogroups L3 and L2 is occupied by two other phylogroups, L4 and L5. Phylogroup L5 is restricted to central Portugal, occupying the region between the Tagus and Douro rivers. L4 has the broadest distribution, occupying the remaining areas of southern Portugal and Spain passing through the Ebro valley to reach the Atlantic and Mediterranean coasts of France.

The root of the network is located along the branch that connects the very divergent lineages L and N, allowing for the inference of the most recent common ancestor (MRCA) for each phylogroup (Figure 3). Although this identification is straightforward for clade N (haplotype 126), the networks reveal two probable ancestral haplotypes within clade L (haplotypes 127 and 104), which are connected to haplotype 126 through a loop. SP and MJ networks constructed with 0-fold degenerate sites only, thus reducing homoplasy within the data set (data not shown) result in the collapse of this reticulation, and haplotype 126 (lineage N) connects unambiguously to haplotype 127 (lineage L), supporting 127 as the ancestral haplotype within phylogroup L. Divergent mitochondrial haplotypes from different phylogroups occurring in sympatry were detected in several populations. The admixed populations were: populations 6, 23, 24 and 25 (phylogroups L2 and L4); populations 15, 17, 18, 19, 20, 21 22 and 96 (phylogroups L4 and L5); population 33 (phylogroups L4 and N); population 44 (phylogroups L1 and L4) and population 56, 58, 78 and 82 (phylogroups L1, L3 and L5) (Additional file 1, Table S1).

**Figure 5:**
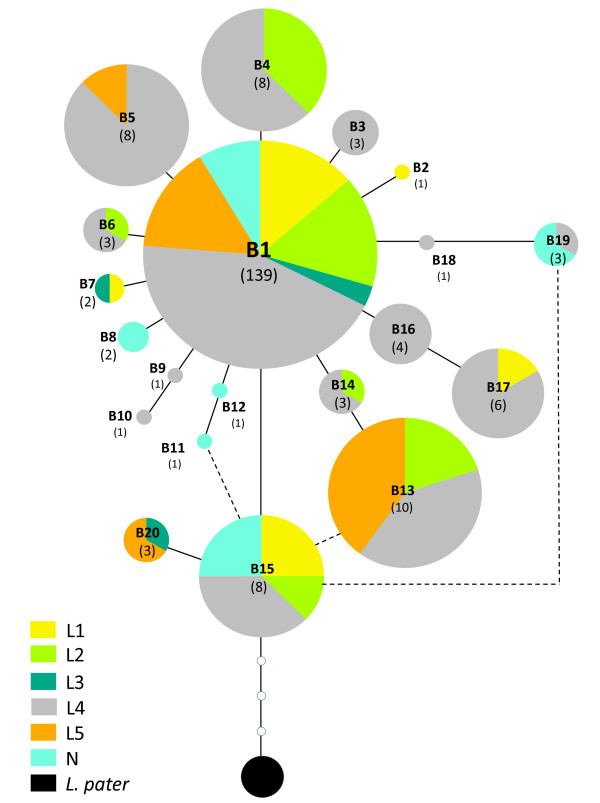
**Statistical Parsimony network of *Lacerta lepida *β-Fibrinogen intron 7 alleles from 104 samples**. Open circles with no numbers represent unsampled or extinct alleles, and the filled black circle represents the outgroup (*Lacerta pater*). Pie chart shading represents the proportion of each allele found within each mitochondrial phylogroup. Colours in pie charts are the same as those used to represent mitochondrial phylogroups in Figure 3. Dashed lines represent ambiguities in the network.

The relationships among *β-fibint7 *haplotypes inferred by the two network construction approaches (MJ and SP) were identical, resulting in a single network with 4 unresolved reticulations (Figure 5). Haplotype B15 is inferred to be the ancestral haplotype as it connects unambiguously to the outgroup. Haplotype B15 is restricted to the south-west of the species distribution (sampling sites 11, 56, 72, 94 and 112). Haplotype B1 is the most common haplotype and has the widest distribution within the group, occurring in 83% of samples. This haplotype is connected to several low frequency haplotypes, generating a star-like genealogy, suggestive of a possible past range expansion and for which signatures of expansion were formally detected by mismatch distribution analysis and neutrality tests (Table 2). Within the 50 southernmost samples, 16 alleles are found, representing 80% of the nuclear allele diversity. From those 16 alleles, 8 are restricted to the southern area.

**Table 2:** Results from mismatch distribution and neutrality tests for *cytb *mtDNA phylogroups and for the *β-fibint7 *nuclear gene.

				Mismatch Distribution				Neutrality tests					
		Spatial genetic structure		Sudden-expansion model		Spatial-expansion model							
				
Locus	Phylogroup	**χ**^**2**^	p	p (SDD)	p (hg)	p (SDD)	p (hg)	*D*	*p*	*Fs*	p	*R2*	p
	L1	73.78	<0.01	0.49	0.67	0.34	0.63	-1.78	0.04	-2.67	0.04	0.12	**0.18**
	L2	358.07	*0.24*	0.15	0.29	0.31	0.36	-1.72	0.02	-9.92	0.00	0.05	0.00
*Cytb*	L3	754.10	<0.01	0.13	0.22	0.28	0.46	-1.18	**0.10**	-7.06	0.00	0.07	**0.09**
	L4	3494.93	*0.09*	0.92	0.67	0.95	0.70	-2.35	0.00	-26.33	0.00	0.02	0.00
	L5	946.18	<0.01	0.10	0.09	0.33	0.33	-2.17	0.00	-26.66	0.00	0.03	0.00
	N	105.14	0.03	0.68	0.78	0.86	0.87	-2.00	0.01	-3.90	0.01	0.06	0.00
β-Fib ^(a)^	...	148.25	0.02	0.76	0.96	0.58	0.85	-1.69	0.01	-18.52	0.00	0.04	0.02
β-Fib ^(b)^	...	-	-	0.74	0.78	0.60	0.81	-1.73	0.01	-18.67	0.00	0.08	0.00

(p(SDD) = sum of square deviations; p(hg) = Harpending's raggedness index; Tajima's D (D) and respective p value; Fu's Fs test (Fs) and respective p value; Ramos-Onsis R2 (R2) and respective p value). Statistics that do not suggest range expansion are shown in bold font. Results for the spatial genetic structure estimated with Geodis are also shown. Statistics that do not show evidence for spatial genetic structure are shown in italic font.

(a) Dataset using haplotypes inferred by Phase with probability threshold > 0.60 (208 haplotypes)

(b) Reduced dataset using haplotypes inferred by Phase with probability threshold > 0.90 (192 haplotypes)

The nuclear data failed to recover the phylogroups detected by the mtDNA dataset. Nevertheless, when the nuclear dataset is grouped according to the mtDNA phylogroups previously identified, structure in the distribution of alleles can be detected. For this analysis each of the mtDNA phylogroups was considered as a geographic region and Geodis was used to test for geographical structure amongst the nuclear genetic variation. Nuclear haplotypes are shared among some of the mtDNA phylogroups (with the exception of phylogroup N): all haplotypes from L3 (B7 and B20) occur either in L1 (B7) or in L5 (B20); all haplotypes from L2 (B4, B6, B13 and B14) occur in L4 (with B13 also occurring in L5); all haplotypes from L5 (B5, B13 and B20) occur either in L4 (B5 and B13), either in L3 (B20) or in L2 (B13) and finally haplotypes from L1 (B7 and B17) occur either in L3 (B7) or in L4 (B17). Additionally all phylogroups share haplotype B1 (the most common and one of the most ancestral haplotypes) and all phylogroups apart from L3 and L5 also share haplotype B15 (the most ancestral haplotype). It is important to note that only geographically close phylogroups share nuclear haplotypes. It is also possible to identify private haplotypes within the geography of several mtDNA phylogroups: B2 was only detected in phylogroup L1; haplotypes B3, B9, B10, B14, B16 and B18 in L4 and haplotypes B8, B11, B12 and B19 in phylogroup N.

### Divergence times

Mean ages and 95% highest posterior density (HPD) of mtDNA phylogroups are shown in Table 3. Divergence within the group is estimated to have started approximately 9.4 million years ago (Ma) (5.58-13.66) in the mid-late Miocene, corresponding to the cladogenetic event between phylogroups N and L. Although divergence within phylogroup L is estimated to have started in the Plio-Pleistocene (1.96 Ma; 1.13-2.91) the current diversity within each phylogroup seems to have Pleistocene origins, with diversification times for all phylogroups estimated to be younger than 1.0 Ma. The oldest split within group L relates to the divergence of the monophyletic lineage composed of phylogroups L2 and L3 from the remaining phylogroups.

**Table 3:** Divergence time estimates in million years (Ma) from the most recent common ancestor (mrca) of all group members from each *Lacerta lepida *phylogroup estimated using the method of Saillard *et al*. (2000) using three different mutation rates; and divergence time estimates for the main nodes recovered in the phylogenetic analysis using a mean mutation rate of 2% in Beast.

		Saillard			Beast		
	Mutation rate	1%	0.85%	1.15%	**1%**^**(a)**^		
mtDNA phylogroups		(mean ± s.d.)			Lower HPD	Mean	Upper HPD
L1		0.64 ± 0.12	0.75 ± 0.14	0.55 ± 0.10	0.28	0.76	1.32
L2		0.45 ± 0.21	0.53 ± 0.24	0.39 ± 0.16	0.21	0.47	0.78
L3		0.59 ± 0.38	0.63 ± 0.40	0.47 ± 0.27	0.32	0.68	1.10
L4		0.92 ± 0.30	1.08 ± 0.35	0.80 ± 0.24	n.a	n.a	n.a
L5		0.59 ± 0.19	0.70 ± 0.22	0.51 ± 0.15	0.29	0.61	0.98
N		0.85 ± 0.34	0.99 ± 0.41	0.74 ± 0.28	0.29	1.04	0.63
L2+L3		n.a	n.a	n.a	0.82	1.50	2.27
L1+L2+L3+L4+L5		n.a	n.a	n.a	1.13	1.96	2.91
All (L+N)		n.a	n.a	n.a	5.58	9.43	13.66

For each node the mean divergence time and the lower and upper values of the highest probability distribution (HPD) are presented. (see text for a detailed explanation of each method).

(a) Divergence times were calculated using mutations rates drawn from a normal distribution with a mean mutation rate of 0.01 mutations/site/million years and a standard deviation of 0.0015.

n.a: not applicable

### Neutrality tests and demographic analyses

**Figure 6:**
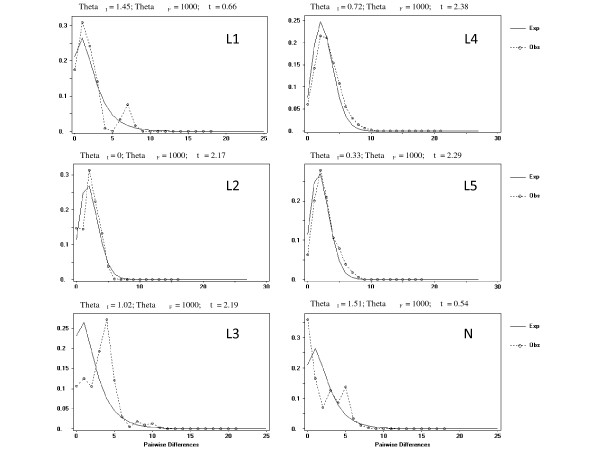
**Mismatch distribution of mtDNA haplotypes for each of the 6 *Lacerta lepida *phylogroups**. The expected frequency is based on a population growth-decline model, determined using DnaSP v4.50 [69] and is represented by a continuous line. The observed frequency is represented by a dotted line.

**Figure 7:**
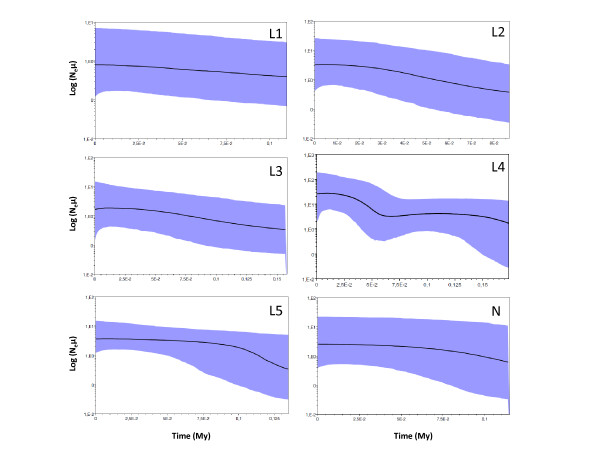
**Bayesian skyline plots showing the historical demographic trends for each *Lacerta lepida *mitochondrial phylogroup detected using cytochrome b sequences**. Along the y-axis is the expressed population size estimated in units of N_e_μ (N_e_: effective population size, μ: mutation rate per haplotype per generation). The y-axis is in a log-scale. Solid lines represent median estimates and shaded areas represent confidence intervals.

Significant deviations from neutrality that could reflect past population expansion events were detected for almost all phylogroups with Tajima's *D*, with the exception of phylogroup L3. When more powerful statistics were applied, a similar pattern of significant deviations from neutrality were observed, with the exception of phylogroups L1 and L3 (Table 2). The distributions of pairwise differences within each phylogroup were also found to be consistent with sudden-expansion and spatial-expansion models (as seen by the *SDD *and *hg *p values in Table 2), with signatures of population growth being exhibited by the bell shaped mismatch distributions (Figure 6). Historical demographic reconstructions (BSPs) also show a trend of population growth in all phylogroups (Figure 7).

### Geographical distribution of alleles and inference of refugial areas

**Figure 8:**
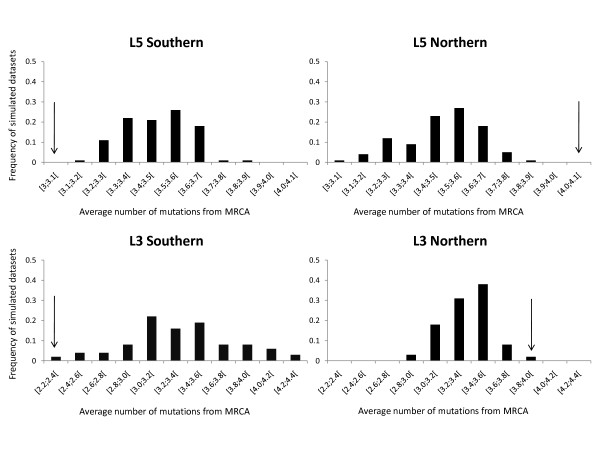
**Observed and null model expected distances of haplotypes from the MRCA in southern and northern regions of phylogroups L3 and L5**. Null model distributions were generated by randomising the geographic states of alleles (see text for details). Arrows indicate observed distances. For both phylogroups the average distance of southern haplotypes from the MRCA is significantly less than would be expected by chance, and the average distance of northern haplotypes from the MRCA is significantly greater than would be expected by chance. L3 southern populations: 78, 81, 82, 83, 85, 93 and 94; L3 northern populations: 87, 89, 90, 91 and 92; L5 southern: 15, 17, 18, 19, 20, 21, 22, 53, 95, 96, 97, 98, 99 and 100; L5 northern populations: 56, 58, 78, 79, 82, 101, 102, 103, 104, 105, 106 and 108.

Within phylogroups, statistically significant associations between genetic variation and geographic distribution were detected for L1, L3, L5 and N (Table 2). More detailed geographic sampling within phylogroups L3 and L5 allowed for the testing of hypotheses of southern refugial origin and northern expansion. The average number of mutations of haplotypes from the MRCA in southern regions of phylogroups L3 and L5 was significantly lower than that expected by chance (2.25 and 3.03 for L3 and L5, respectively) (Figure 8). In contrast, northern haplotypes exhibit a significantly higher average number of mutations from the MRCA than expected by chance (3.85 and 4.04 for L3 and L5, respectively) (Figure 8). These results reveal significant associations of ancestral haplotypes with southern ranges and derived haplotypes with northern regions for these two phylogroups.

## Discussion

Detailed analysis of the distribution of mitochondrial genetic variation within *Lacerta lepida *across the Iberian Peninsula revealed a complex phylogeographic history for the species. The *cytb *genealogy clearly defines 6 geographic phylogroups with generally non-overlapping geographic distributions. The strong association of mtDNA genetic variation with geography suggests a history of allopatric divergence in different refugia within the Iberian Peninsula, a pattern that has been described for several taxa within the region [see [5], and references therein]. Although this pattern of differentiation of distinct evolutionary units in allopatry was less evident from the analysis of the nuclear data, the distribution of nuclear haplotypes is not in conflict with the mtDNA phylogroups. Failure of the nuclear gene genealogy to reveal concordant genetic structure with the mitochondrial genealogy can be expected if one takes into account the fact that nuclear genes take on average four times longer to reach monophyly than mitochondrial ones [73]. In fact, most of the intraspecific differentiation within *Lacerta lepida *is of relatively recent origin, with the majority of phylogroups (L1 to L5) estimated to have diversified within the Pleistocene, increasing the probability of mitochondrial lineages not being reciprocally monophyletic for nuclear markers. Nevertheless, phylogroup N is estimated to have diverged from the remaining phylogroups during the Miocene (5.6-13.7 Ma) representing a much older cladogenetic event within the group. This older split therefore allows for more time for lineage sorting at the nuclear level. This is evident in the composition of nuclear haplotypes of phylogroup N, which are almost all private with the exception of the ancestral haplotypes (which are shared among almost all phylogroups). Thus, although mtDNA lineages have not reached monophyly at the nuclear level, some level of nuclear differentiation between mtDNA lineages is detected by the existence of private alleles, and this is most pronounced for phylogroups that represent older cladogenetic events (L1, L4 and N). Incomplete lineage sorting seems therefore a plausible explanation for the discrepancies in mitochondrial and nuclear gene genealogies. Recent phylogeographic and phylogenetic studies focusing on lizards in the Iberian Peninsula reveal similar discrepant patterns between mtDNA and nuclear genealogies [10,13]. In the case of *Podarcis *wall lizards Pinho *et al*. [13] showed that, despite a complete lack of monophyly at the nuclear level, most of the species show historical reproductive isolation and that the lack of monophyly is mainly a result of shared ancestral polymorphism.

Although it is plausible that incomplete lineage sorting is responsible for the failure of nuclear genealogy to recover the mitochondrial phylogroups in *Lacerta lepida*, current gene flow, mainly male-biased, at zones of secondary contact can also be invoked to explain the patterns observed. Evidence for this comes from the observation that geographically close phylogroups share more derived haplotypes. For example allele B20 occurs only in the very divergent phylogroups L3 and L5 near the zone of contact, but it was not detected in the ancestral phylogroup L4, suggesting that nuclear gene flow may be occurring between the L3 and L5 mtDNA lineages. The same is true for allele B4 which, although more widespread within phylogroup L4, is also found in individuals of L2 near the zone of contact, but was not detected in phylogroup L3, which is phylogenetically closer to phylogroup L2.

### Historical biogeography of *Lacerta lepida*

Divergence within *Lacerta lepida *is estimated to have started in the Miocene, approximately 9.4 Mya (5.58-13.66), with divergence of phylogroup N from phylogroup L. Within phylogroup L estimated divergence times are much younger and inferred to have been initiated in the Plio-Pleistocene, approximately 1.96 Mya (1.13-2.91 Mya). Interestingly, although divergences between the mitochondrial phylogroups have a Plio-Pleistocene (within group L) or a late Miocene (for group N) origin, haplotype diversities within phylogroups indicate a strong influence of later Pleistocene events, with diversification within phylogroups starting between 0.92 and 0.45 Ma. The importance of the Pleistocene climatic oscillations in promoting species differentiation in the Iberian Peninsula has been emphasized by previous studies [see [5], for a recent review] and this clearly also seems to be the case for *Lacerta lepida*.

#### Phylogroup N

Phylogroup N is distributed across the Betic Mountain range in south-western Spain and its distribution approximately coincides with the described subspecies *Lacerta lepida nevadensis*. The existence of clear significant morphological differentiation between *L*. *l*. *nevadensis *and the remaining subspecies of *Lacerta lepida *[74-76], together with high levels of allozyme differentiation [76] and mitochondrial genetic differentiation (our study and [17]), suggests that the two lineages (N and L) might in fact reflect two different species. Paulo *et al*. [17] have inferred that divergence between phylogroups L and N were initiated by overseas dispersal between what was then the Iberian mainland and the Betic Massif that at that time existed as an island between Iberia and North Africa. Under this scenario subsequent contact between the phylogroups would have been initiated after the merging of the Betic Massif with Iberian mainland, due to the closing of the Betic corridor 7.8-7.6 Ma [see [77,78] for a detailed explanation of the kinematics of the western Mediterranean basin]. The Betic Mountain range is a region of high endemism for plants and animals, and its importance as a refugium for other taxa has been highlighted before [see [5]]. Interestingly, some of these taxa show similar divergence times and distribution as phylogroup N (e.g. *Salamandra salamandra longirostris *[79] and *Alytes dickhilleni *[80]).

#### Phylogroups L2 and L3

The monophyletic group composed of phylogroups L2 and L3 is estimated to have started diverging from the remaining phylogroups in the early Pleistocene, approximately 1.5 Mya (0.82-2.27). Interestingly these two phylogroups have a disjunct distribution, with phylogroup L2 occupying the south of Portugal whereas L3 occupies the north-western parts of the Iberian Peninsula. The intervening region between phylogroups L2 and L3 is occupied by phylogroup L5. A vicariant event during the Middle Pleistocene (0.82-2.27 Mya) triggering divergence between the L2-L3 lineage and the remaining populations of *Lacerta lepida *seems probable. It is noteworthy that most phylogeographic studies within Iberia reveal similar phylogenetic breaks associated with the same period (e.g. *Chioglossa lusitanica *[6,7], *Oryctolagus cuniculus *[81], *Lacerta schreiberi *[9,82]), suggesting a common vicariant history. Such a vicariant event was most likely climatically-mediated as no apparent geographical barrier exists within western Iberian Peninsula.

The western Algarve region in southern Portugal has been indicated as the evolutionary centre for several species and also as a key refugial area [83,84]. The region harboured relicts of temperate forests during the Last Glacial Maximum [85], probably providing suitable conditions for species survival through glacial periods. The high genetic distance and disjunct distribution found between phylogroups L2 and L3 is most likely the consequence of fragmentation of a once more continuous range in response to a cooling climate. Subsequent climate amelioration during an interglacial period probably resulted in this distribution gap being colonized by L5, expanding from another nearby refugium. A similar distribution pattern of genetic variation is found within *Discoglossus galganoi *across Portugal, with two closely related phylogroups distributed in the south and north and a less related phylogroup bisecting their distribution [86]. The distribution of *Discoglossus galganoi *phylogroups and the evolutionary relationship between them is remarkably similar to that found within *Lacerta lepida*.

#### Phylogroups L1, L4 and L5

The refugia for *Lacerta lepida *phylogroups L1, L4 and L5 were probably located in the south-eastern side of the Guadalquivir basin. Support for this comes from the distribution of ancestral haplotypes 127, 140 and 8 within phylogroup L4 that are found only in this region (localities 10, 12, 32, 36, 37, 38, 39, 40, 41). The two very divergent haplotypes within L4 (60 and 61) are also restricted to this region. The widespread distribution of the most frequently sampled haplotype 1 suggests that the spatial and demographic expansion detected within L4 was of a "leading edge" type [87,88], with few individuals rapidly colonizing adjacent regions, leading to a decrease in genetic diversity on the newly colonized areas. The different phylogroups are most likely the result of three different expansions from the southern refugia dominated by different ancestral haplotypes, followed by further divergence in allopatry.

### Refugial areas, range expansions, and secondary contact

The lower mutation rate and slower fixation rate of nuclear genes, when compared to the mitochondrial genome, mean that nuclear genealogies may be more indicative of older demographic events [89]. Therefore, the analysis of the distribution of ancestral nuclear alleles can be useful in the identification of refugial areas. Haplotype B15 is the root of the nuclear gene network, representing the most ancestral allele in the dataset. The distribution of B15 is primarily in the southern region of Iberia, where it occurs with higher frequency than in northern regions (70% of samples with haplotype B15 are from southern latitudes). Furthermore the southern region of Iberia (to the south of river Tagus) shows higher nuclear haplotype richness, with 90% of the identified haplotypes occurring in this region from which 67% do not occur further north. The high nuclear haplotype richness found in the southern regions of Iberia, together with the high frequency of B15 in this region suggest that southern populations are older and the source of subsequent northern expansions.

The geographical and genealogical relationships of mitochondrial haplotypes within phylogroups provide further indications as to the probable refugial areas from where range expansions subsequently occurred. The same pattern of older (interior) haplotypes being typically found within southern sampling sites is also evident for the mitochondrial dataset. For example within phylogroup L2, the ancestral haplotype 96 and the related interior haplotype 93 are most frequent in southern sites 25, 63, 64, 66, 69 and 70, coincident with the Algarve and southern Alentejo region in Portugal. Within phylogroup L3 the ancestral haplotype 51 and the related interior haplotype 46 occur either in the southern region of the phylogroup distribution, to the south of Douro river (sites 82 and 78), or in sites located at the centre of the phylogroup distribution (sites 87, 89 and 92). Within phylogroup L4, the ancestral haplotype 127 and the closely related haplotypes 140 and 8 occur again only in the south-eastern limit of this phylogroup (sites 10, 12, 32, 36, 37, 38, 39, 40 and 41), near the source of the Guadalquivir river in Jaen, Spain. Finally the ancestral haplotype of phylogroup L5 (haplotype 13) and the related interior haplotypes 14 and 17 are most frequent within southern sites of this phylogroup (sites 22, 95, 96 and 99), along the river Tagus basin. These patterns are statistically significant for phylogroups L3 and L5 (Figure 8), which also reveal an excess of derived haplotypes in the north of their geographic ranges. These repeated pattern of ancestral haplotypes occurring more frequently towards the southern range of phylogroups, and in some cases derived haplotypes being more frequent within more northern sites, suggests that southern regions within phylogroup ranges have probably functioned as refugia during past adverse climatic conditions, from which subsequent northern expansions occurred.

The evolutionary history of *Lacerta lepida *is consistent with a history of population fragmentation and allopatric divergence in southern refugia, followed by recent demographic and spatial range expansions. Demographic expansions are supported by the neutrality and mismatch distribution tests that reveal signatures of demographic and spatial expansions in almost all phylogroups and by the BSP analysis, which show a trend of population expansion in all phylogroups between 0.1 to 0.15 million years ago (Figure 7). Spatial expansions are supported by the latitudinal variation observed for the distributions of ancestral and derived haplotypes. This history of diversification in allopatry followed by range expansions has lead to the establishment of at least four secondary contact zones where very divergent mitochondrial haplotypes belonging to different phylogroups were found in sympatry (Figure 4). Further analyses of these zones may provide insights into the mechanisms involved in speciation and divergence within this lizard species.

## Conclusions

Mitochondrial and nuclear gene genealogies in *Lacerta lepida *provide evidence for a history of isolation and divergence in allopatry resulting in the diversification of six genetically and geographically distinct lineages. Although diversification within the group is largely concordant with the onset of the major glaciations at the beginning of the Pleistocene (approximately 2 Mya), an earlier event associated with the Miocene was also identified. This event (approximately 9 Mya), which marks the divergence of lineages N and L, seems to be associated with geological events related to the evolution of the Mediterranean basin. Signatures of recent demographic and spatial expansions were apparent in all phylogroups, with several phylogroups having established zones of secondary contact. Analyses of the distribution of ancestral and derived alleles within each phylogroup, and inferences related to the biogeography of *Lacerta lepida*, allowed the identification of probable refugia within the Iberian Peninsula, suggesting southern refugial areas within phylogroups. Our work highlights the importance of these areas for the long-term conservation and management of diversity with *Lacerta lepida *across its geographic range.

## Authors' contributions

AM conceived the study, carried out fieldwork, molecular genetic work, data analysis and drafted the manuscript. OSP participated in the study design and helped to draft the manuscript. BCE and GMH participated in the study design, helped to draft the manuscript and coordinated the overall development of the study. All authors read and approved the final manuscript.

## Supplementary Material

Additional file 1 **Table S1**. This file includes one table (Table S1) with information about sampling localities, number of samples per locality and haplotypes detected in each locality. Click here for file
