# Supplementary material for: Phylogeography and demographic history of Lacerta lepida in the Iberian Peninsula: multiple refugia, range expansions and secondary contact zones
Source: BMC Evol Biol. 2011 Jun 17;11:170. doi: 10.1186/1471-2148-11-170 (PMC3141430; doi:10.1186/1471-2148-11-170)
Supplement: Additional file 1 — Table S1. This file includes one table (Table S1) with information about sampling localities, number of samples per locality and haplotypes detected in each locality. [file 1471-2148-11-170-S1.DOC]

Table A1 Number of samples per site (n) and correspondent number of sequences successfully amplified for *cytb* and β-Fibrinogen intron 7 (*β-Fibint7*) genes. For each site the haplotypes found for each gene are shown.

|  |  | **N^o^ of sequences** | | |  |  | | **mtDNA** |
| --- | --- | --- | --- | --- | --- | --- | --- | --- |
| **Site** | **n** |  | ***Cytb*** | ***β-Fibint7*** |  | ***Cytb* haplotypes** | ***β-Fib* alleles** | **Phylogroup** |
| 1 | 2 |  | 2 | 2 |  | 1 | B13, B14 | L4 |
| 2 | 1 |  | 1 | 0 |  | 7 | n.a. | L4 |
| 3 | 1 |  | 1 | 1 |  | 2 | B1 | L4 |
| 4 | 1 |  | 1 | 0 |  | 1 | n.a. | L4 |
| 5 | 3 |  | 3 | 0 |  | 1, 66, 83 | n.a. | L4 |
| 6 | 3 |  | 3 | 1 |  | 6, 63, 97 | B1, B4 | L2, L4 |
| 7 | 3 |  | 3 | 0 |  | 1, 62, 63 | n.a. | L4 |
| 8 | 3 |  | 3 | 3 |  | 63, 77, 136 | B1, B3^†^, B4^†^ B13 | L4 |
| 9 | 2 |  | 2 | 2 |  | 10, 78 | B1, B6 | L4 |
| 10 | 1 |  | 1 | 0 |  | 8 | n.a. | L4 |
| 11 | 5 |  | 4 | 4 |  | 10, 63, 86 | B1, B3, B15^†^, B16^†^ | L4 |
| 12 | 1 |  | 1 | 0 |  | 8 | n.a. | L4 |
| 13 | 1 |  | 1 | 0 |  | 73 | n.a. | L4 |
| 14 | 3 |  | 3 | 0 |  | 1 | n.a. | L4 |
| 15 | 5 |  | 5 | 0 |  | 1, 25, 72, 76 | n.a. | L4, L5 |
| 16 | 3 |  | 3 | 0 |  | 62 | n.a. | L4 |
| 17 | 9 |  | 9 | 0 |  | 1, 6, 16, 62, 79, 114 | n.a. | L4, L5 |
| 18 | 6 |  | 6 | 0 |  | 1, 25, 62,75 | n.a. | L4, L5 |
| 19 | 5 |  | 5 | 1 |  | 25, 67, 72, 80 | B1 | L4, L5 |
| 20 | 2 |  | 2 | 0 |  | 25, 65 | n.a. | L4, L5 |
| 21 | 5 |  | 5 | 0 |  | 1, 25, 72 | n.a. | L4, L5 |
| 22 | 5 |  | 5 | 2 |  | 1, 17, 15, 109, 111 | B1, B13 | L4, L5 |
| 23 | 3 |  | 3 | 0 |  | 100, 113, 118 | n.a. | L2, L4 |
| 24 | 9 |  | 9 | 0 |  | 6, 68, 69, 74, 81, 91, 110, 112 | n.a. | L2, L4 |
| 25 | 8 |  | 8 | 2 |  | 63, 71, 91, 93 | B1, B13, B14 | L2, L4 |
| 26 | 3 |  | 3 | 3 |  | 128, 132, 135 | B1, B5, B16 | L4 |
|  |  | **N^o^ of sequences** | | |  |  | | **mtDNA** |
| **Site** | **n** |  | ***Cytb*** | ***β-Fibint7*** |  | ***Cytb* haplotypes** | ***β-Fibint7* alleles** | **Phylogroup** |
| 27 | 5 |  | 4 | 4 |  | 10, 84, 130 | B1, B5 | L4 |
| 28 | 1 |  | 1 | 0 |  | 60 | n.a. | L4 |
| 29 | 5 |  | 2 | 5 |  | 133, 134 | B1, B4, B5, B13 | L4 |
| 30 | 2 |  | 2 | 0 |  | 9, 82 | n.a. | L4 |
| 31 | 4 |  | 3 | 3 |  | 129, 133, 134 | B1, B4, B18 | L4 |
| 32 | 1 |  | 1 | 0 |  | 8 | n.a. | L4 |
| 33 | 2 |  | 2 | 0 |  | 85, 119 | n.a. | L4, N |
| 34 | 1 |  | 1 | 1 |  | 82 | B1 | L4 |
| 35 | 2 |  | 1 | 1 |  | 82 | B1, B9 | L4 |
| 36 | 4 |  | 4 | 0 |  | 82, 140, 141 | n.a. | L4 |
| 37 | 6 |  | 6 | 0 |  | 8, 82, 87, 142 | n.a. | L4 |
| 38 | 3 |  | 3 | 0 |  | 8, 144, 145 | n.a. | L4 |
| 39 | 4 |  | 4 | 0 |  | 8, 140, 153 | n.a. | L4 |
| 40 | 3 |  | 3 | 1 |  | 3, 5, 8 | B1, B17 | L4 |
| 41 | 1 |  | 1 | 0 |  | 127 | n.a. | L4 |
| 42 | 1 |  | 1 | 0 |  | 1 | n.a. | L4 |
| 43 | 3 |  | 3 | 1 |  | 1, 11, 12 | B1^†^, B19^†^ | L4 |
| 44 | 3 |  | 3 | 0 |  | 1, 108 | n.a. | L1, L4 |
| 45 | 1 |  | 1 | 0 |  | 62 | n.a. | L4 |
| 46 | 3 |  | 3 | 2 |  | 1, 62 | B1 | L4 |
| 47 | 3 |  | 3 | 3 |  | 1, 64 | B1, B17 | L4 |
| 48 | 2 |  | 2 | 0 |  | 1 | n.a. | L4 |
| 49 | 3 |  | 3 | 0 |  | 1 | n.a. | L4 |
| 50 | 1 |  | 1 | 0 |  | 4 | n.a. | L4 |
| 51 | 3 |  | 3 | 0 |  | 85, 139 | n.a. | L4 |
| 52 | 1 |  | 1 | 0 |  | 1 | n.a. | L4 |
| 53 | 3 |  | 3 | 2 |  | 25, 70, 90 | B1 | L4, L5 |
|  |  | **N^o^ of sequences** | | |  |  | | **mtDNA** |
| **Site** | **n** |  | ***Cytb*** | ***β-Fibint7*** |  | ***Cytb* haplotypes** | ***β-Fibint7* alleles** | **Phylogroup** |
| 54 | 6 |  | 6 | 3 |  | 104, 105 | B1 | L1 |
| 55 | 2 |  | 2 | 1 |  | 104, 106 | B1 | L1 |
| 56 | 5 |  | 5 | 1 |  | 27, 56, 57 | B1, B15 | L1, L5 |
| 57 | 1 |  | 1 | 0 |  | 58 | n.a. | L1 |
| 58 | 6 |  | 6 | 2 |  | 31, 34, 37, 38, 56 | B1 | L1, L5 |
| 59 | 2 |  | 1* | 1 |  | 59, * | B1 | L1 |
| 60 | 1 |  | 1 | 1 |  | 61 | B1, B17 | L1 |
| 61 | 4 |  | 4 | 1 |  | 91, 97, 99, 101 | B1 | L2 |
| 62 | 5 |  | 5 | 0 |  | 91, 115 | n.a. | L2 |
| 63 | 1 |  | 1 | 0 |  | 93 | n.a. | L2 |
| 64 | 3 |  | 3 | 1 |  | 93, 97, 116 | B4 | L2 |
| 65 | 1 |  | 1 | 1 |  | 94 | B1 | L2 |
| 66 | 3 |  | 3 | 1 |  | 93, 117 | B1 | L2 |
| 67 | 1 |  | 1 | 0 |  | 91 | n.a. | L2 |
| 68 | 1 |  | 1 | 1 |  | 91 | B1 | L2 |
| 69 | 1 |  | 1 | 1 |  | 96 | B1, B13 | L2 |
| 70 | 2 |  | 2 | 0 |  | 93, 96 | n.a. | L2 |
| 71 | 1 |  | 1 | 1 |  | 103 | B1 | L2 |
| 72 | 6 |  | 6 | 2 |  | 91, 93, 94, 95, 98 | B1, B15 | L2 |
| 73 | 1 |  | 1 | 0 |  | 100 | n.a. | L2 |
| 74 | 1 |  | 1 | 1 |  | 102 | B1 | L2 |
| 75 | 1 |  | 1 | 1 |  | 93 | B1, B6 | L2 |
| 76 | 1 |  | 1 | 0 |  | 93 | n.a. | L2 |
| 77 | 1 |  | 1 | 1 |  | 92 | B1 | L2 |
| 78 | 2 |  | 2 | 0 |  | 32, 46 | n.a. | L3, L5 |
| 79 | 3* |  | 1 | 0 |  | 25 | n.a. | L5 |
| 80 | 1* |  | 0 | 0 |  | n.a. | n.a. | n.a. |
|  |  | **N^o^ of sequences** | | |  |  | | **mtDNA** |
| **Site** | **n** |  | ***Cytb*** | ***β-Fibint7*** |  | ***Cytb* haplotypes** | ***β-Fibint7* alleles** | **Phylogroup** |
| 81 | 1 |  | 1 | 0 |  | 40 | n.a. | L3 |
| 82 | 4 |  | 4 | 0 |  | 17, 21, 46, 48 | n.a. | L3, L5 |
| 83 | 1 |  | 1 | 0 |  | 54 | n.a. | L3 |
| 84 | 1* |  | 0 | 0 |  | n.a. | n.a. | n.a. |
| 85 | 1 |  | 1 | 0 |  | 47 | n.a. | L3 |
| 86 | 2* |  | 0 | 0 |  | n.a. | n.a. | n.a. |
| 87 | 3 |  | 3 | 0 |  | 42, 52, 53 | n.a. | L3 |
| 88 | 1* |  | 0 | 0 |  | n.a. | n.a. | n.a |
| 89 | 10* |  | 9 | 0 |  | 41, 44, 45, 46, 50, 51, 55* | n.a. | L3 |
| 90 | 11 |  | 11 | 1 |  | 41, 43 | B1 | L3 |
| 91 | 1 |  | 1 | 0 |  | 42 | n.a. | L3 |
| 92 | 3 |  | 3 | 1 |  | 41, 46, 48 | B1 | L3 |
| 93 | 5 |  | 5 | 0 |  | 49 | n.a. | L3 |
| 94 | 4 |  | 1 | 3 |  | 131 | B1, B2, B7^†^, B15^†^ | L3 |
| 95 | 3 |  | 3 | 1 |  | 14, 17, 20 | B1 | L5 |
| 96 | 2 |  | 2 | 0 |  | 13, 76 | n.a. | L4, L5 |
| 97 | 1 |  | 1 | 0 |  | 15 | n.a. | L5 |
| 98 | 1 |  | 1 | 0 |  | 18 | n.a. | L5 |
| 99 | 8 |  | 8 | 2 |  | 17, 33, 88, 89 | B1, B20 | L5 |
| 100 | 2 |  | 2 | 0 |  | 30 | n.a. | L5 |
| 101 | 7 |  | 7 | 3 |  | 23, 26, 29, 30 | B1, B5 | L5 |
| 102 | 1 |  | 1 | 1 |  | 17 | B1 | L5 |
| 103 | 2 |  | 2 | 1 |  | 19, 32 | B1 | L5 |
| 104 | 1 |  | 1 | 0 |  | 35 | n.a. | L5 |
| 105 | 1 |  | 1 | 1 |  | 24 | B13, B20 | L5 |
| 106 | 3 |  | 3 | 0 |  | 28, 36, 39 | n.a. | L5 |
| 107 | 1 |  | 1 | 0 |  | 107 | n.a. | L4 |
|  |  | **N^o^ of sequences** | | |  |  | | **mtDNA** |
| **Site** | **n** |  | ***Cytb*** | ***β-Fibint7*** |  | ***Cytb* haplotypes** | ***β-Fibint7*alleles** | **Phylogroup** |
| 108 | 1 |  | 1 | 1 |  | 22 | B1 | L5 |
| 109 | 1 |  | 1 | 0 |  | 126 | n.a. | N |
| 110 | 1 |  | 1 | 0 |  | 124 | n.a. | N |
| 111 | 5 |  | 4 | 1 |  | 120, 121, 123, 125 | B1, B8 | N |
| 112 | 11 |  | 6 | 7 |  | 119 | B1^†^,B8, B11, B12, B15, B19^†^ | N |
| 113 | 1 |  | 1 | 0 |  | 122 | n.a. | N |
| 114 | 3 |  | 3 | 0 |  | 119 | n.a. | N |
| 115 | 3 |  | 3 | 0 |  | 119, 137 | n.a. | N |
| 116 | 3 |  | 3 | 0 |  | 119, 138 | n.a. | N |
| 117 | 1 |  | 0 | 1 |  | n.a. | B7^†^, B20^†^ | L3+ |
| 118 | 3 |  | 0 | 3 |  | n.a. | B1 | L4+ |
| 119 | 1 |  | 0 | 1 |  | n.a. | B1, B17 | L4+ |
| 120 | 1 |  | 0 | 1 |  | n.a. | B1, B17 | L4+ |
| 121 | 1 |  | 0 | 1 |  | n.a. | B1, B 17 | L4+ |
| 122 | 1 |  | 0 | 1 |  | n.a. | B1 | L4+ |
| 123 | 2 |  | 0 | 2 |  | n.a. | B1 | L4+ |
| 124 | 1 |  | 0 | 1 |  | n.a. | B1, B10 | L4+ |
| 125 | 1 |  | 0 | 1 |  | n.a. | B4^†^, B5^†^ | L4+ |
| 126 | 1 |  | 0 | 1 |  | n.a. | B1, B5 | L4+ |
| 127 | 1 |  | 0 | 1 |  | n.a. | B1 | N+ |
| 128 | 1 |  | 0 | 1 |  | n.a. | B1 | N+ |
| 129 | 1 |  | 0 | 1 |  | n.a. | B5^†^, B6^†^ | L4+ |

* Sampling site with at least one polymorphic sample for *cytb*; † Haplotypes inferred with PHASE probability threshold between 0.60 and 0.90; + Phylogroup was inferred using the geographic location of samples; n.a. information not available.
